# Isolation of Betulinic Acid, its Methyl Ester and Guaiane Sesquiterpenoids with Protein Tyrosine Phosphatase 1B Inhibitory Activity from the Roots of *Saussurea lappa* C.B.Clarke

**DOI:** 10.3390/molecules14010266

**Published:** 2008-01-08

**Authors:** Ji Young Choi, MinKyun Na, In Hyun Hwang, Seung Ho Lee, Eun Young Bae, Bo Yeon Kim, Jong Seog Ahn

**Affiliations:** 1 College of Pharmacy, Yeungnam University, Gyeongsan, Gyeongbuk 712-749, Korea; 2 Korea Research Institute of Bioscience and Biotechnology (KRIBB), 52 Eoun-dong, Yuseong-gu, Daejeon 305-806, Korea

**Keywords:** Protein tyrosine phosphatase 1B, *Saussurea lappa* C.B.Clarke, Betulinic acid, Betulinic acid methyl ester, Mokko lactone, Dehydrocostuslactone.

## Abstract

Activity-guided fractionation of a MeOH extract of the roots of *Saussurea lappa* C.B.Clarke (Compositae), using an *in vitro* protein tyrosine phosphatase 1B (PTP1B) inhibition assay, led to the isolation of four active constituents: betulinic acid (**1**), betulinic acid methyl ester (**2**), mokko lactone (**3**) and dehydrocostuslactone (**4**), along with nine inactive compounds. Our findings indicate that betulinic acid (**1**) and its methyl ester **2**, as well as the two guaiane sesquiterpenoids **3** and **4** are potential lead moieties for the development of new PTP1B inhibitors.

## Introduction

Protein tyrosine phosphatases (PTPases) are expressed in insulin-sensitive tissues (such as liver, muscle and adipose tissue) and have a key role in the regulation of insulin signal transduction pathway [1]. PTP1B, one of the PTPases, is known to be a negative regulator of insulin signal transduction by dephosphorylating the insulin receptor (IR) as well as its substrate, insulin receptor substrates [2,3]. Consequently, the PTP1B inhibitors are recognized as potential therapeutic agents for the treatment of type II diabetes and obesity [4]. As part of our ongoing study to search for new types of PTP1B inhibitors, we found out that a MeOH extract of the roots of *Saussurea lappa* C.B.Clarke (Compositae) inhibited the enzyme activity at a level of 30 μg/mL. The roots of *S. lappa* have been traditionally used as an ethnomedicine to treat gastric pain, abdominal pain, distension, lack of appetite, anorexia, nausea and vomiting [5, 6]. Sesquiterpenes, triterpenes, alkaloids, lignans, and tannins have been reported as constituents of this species [7,8,9,10,11,12,13]. Pharmacological studies on the plant revealed that it has anti-ulcer [8, 9], anti-carcinogenesis [10, 11], and vasorelaxant effects [12], and inhibit nitric oxide (NO) production in lipopolysaccharide (LPS)-activated mouse model [13]. We also reported that some of the isolates from *S. lappa* inhibited the 3-isobutyl-1-methylxanthine (IBMX)-induced melanogenesis in B-16 mouse melanoma cells [7]. Interestingly, there was a report on the isolation of PTP1B inhibitory constituents from *S. lappa*, and Li *et al*. reported that anthraquinones isolated from its roots had moderate PTP1B inhibitory activities [14]. However, in our current investigation, we found out that betulinic acid (**1**) and its methyl ester **2**, as well as two guaiane sesquiterpenoids, mokko lactone (**3**) and dehydrocostuslactone (**4**), had a high PTP1B inhibitory activity. In this paper, we describe the bioassay-guided isolation of active compounds, and their PTP1B inhibitory activity.

## Results and Discussion

Through bioassay-guided fractionation of a MeOH extract of the roots of *S. lappa* 13 compounds were obtained. All the compounds isolated were identified as known constituents of this species [7]. The assay for PTP1B inhibitory activity of all of them, as shown in table 1, revealed that the compounds **1**, **2**, **3**, and **4** inhibited PTP1B activity with 0.70 ± 0.03, 0.93 ± 0.07, 1.41 ± 0.02, and 6.51 ± 0.64 μg/mL, respectively. In particular, betulinic acid (**1**) and its methyl ester **2** displayed the highest activity, which was comparable to those of ursolic acid and RK-682 used as positive controls. However, compounds **5** **–** **13** did not show PTP1B inhibitory activity in our enzyme assay system (less than 50% inhibition at the level of 30 μg/mL). Only two guaiane-type sesquiterpenoids showed the PTP1B inhibitory activity, while eudesmane-type and germacrane-type sesquiterpenoids appeared to be inactive. 

Recently, betulinic acid isolated from *Alnus hirsuta* Ruprecht was demonstrated to inhibit diacylglycerol acyltransferase (DGAT) activity so that it was proposed as a lead moiety for the development of drug for metabolic diseases such as diabetes and obesity [15]. In addition, guaiane sesquiterpenoids, zaluzanin c and 9α-hydroxyguaian-4(15),10(14),11(13)-triene-6,12-olide, isolated from the roots of *Ixeris dentata* forma *albiflora* Nakai were reported to have DGAT inhibitory activity [16]. Although betulinic acid is a common compound widely distributed in many plant species, it attracts our attention because of its various biological activities [17]. On the basis of our findings, we suggest that betulinic acid derivatives and guaiane sesquiterpenoids are capable of inhibiting PTP1B activity. These-type molecules are known to be favorable to cellular penetration, which increase the potential use in the development of new PTP1B inhibitors, as the target is intracellular. 

**Figure 1:**
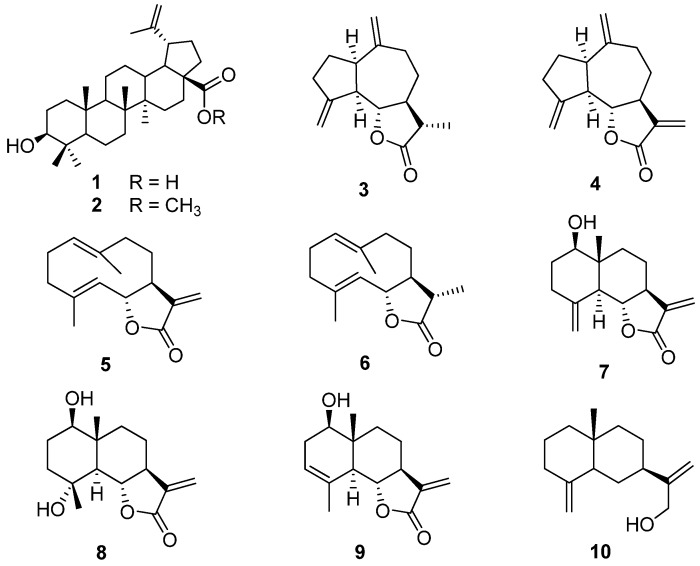
Chemical structures of compounds isolated from the roots of *S. lappa.*

**Table 1:** PTP1B inhibitory activity of compounds **1** – **13** isolated from the roots of *S. lappa*

Compound	Inhibition*^a^* %	IC_50_ (μg/mL)
MeOH extract	61.0	
EtOAc fraction	84.0	
H_2_O fraction	22.3	
Betulinic acid (**1**)	95.1	0.7 ± 0.03
Betulinic acid methyl ester (**2**)	89.4	0.9 ± 0.07
Mokko lactone (**3**)	93.1	1.4 ± 0.02
Dehydrocostuslactone (**4**)	86.2	6.5 ± 0.64
Costunolide (**5**)	< 50.0	na*^b^*
11β,13-Dihydrocostunolide (**6**)	< 50.0	na
Reynosin (**7**)	< 50.0	na
1β-Hydroxy arbusculin A (**8**)	< 50.0	na
Santamarine (**9**)	< 50.0	na
α-Costol (**10**)	< 50.0	na
β-Sitosterol (**11**)	< 50.0	na
Aplotaxene (**12**)	< 50.0	na
α-Amyrin stearate (**13**)	< 50.0	na
Ursolic acid*^c^*	99.0	0.7 ± 0.05
RK-682*^c^*	98.5	1.2 ± 0.09

*^a^* Inhibition (%) of PTP1B activity at 30 μg/mL; *^b^* na: not active (less than 50% inhibition at the level of 30 μg/mL); *^c^* Positive controls

## Experimental

### General

Optical rotations were measured using a JASCO DIP-1000 (Tokyo, Japan) automatic digital polarimeter. The FT-IR spectra were recorded on a JASCO FT-IR 300E spectrophotometer, and UV spectra on a JASCO V-550 spectrophotometer. The NMR spectra were recorded on Bruker 250 MHz (DMX 250) spectrometer, using Bruker's standard pulse program, with chemical shifts reported in ppm downfield from TMS. The EI-MS spectra were recorded on a Micromass spectrum (AUTOSPEC, UK). Column chromatography was carried out on Merck silica gel (70-230 mesh, Merck), MCI-gel CHP-20P (75-150 μm, Mitsubishi Chem. Co.) and Merck Lichroprep RP-18 gel (40-63 μm). TLC was performed on aluminum plates precoated with Kieselgel 60 F_254_ (Merck). All other chemicals and solvents were of analytical grade.

### Plant material

Dried roots of *S. lappa* (Compositae) were purchased in March 2006 from a folk medicine market (“Yak-ryong-si”) in Daegu, Korea, and identified by Prof. See Ryun Chung (Yeungnam University, Korea). A voucher specimen (SH006-179) has been deposited at the College of Pharmacy, Yeungnam University, Korea. 

### Bioassay-guided fractionation of the MeOH extract

The roots of *S. lappa* were extracted three times with MeOH at room temperature for seven days. The MeOH extract that showed about 61% PTP1B inhibitory activity at 30 μg/mL was suspended in water and partitioned with EtOAc, and the EtOAc-soluble fraction displayed higher activity (about 84% inhibitory activity at 30 μg/mL) than that of original extract. From the EtOAc-soluble fraction, 13 subfractions (SEfr. 1 ~ 13) were obtained by silica gel chromatography eluting with a CH_2_Cl_2_-MeOH gradient from 100:0 to 0:100. Of the fractions obtained, SEfr. 4, 5, 6, and 7 showed strong PTP1B inhibitory activity (over than 80% inhibition at 30 μg/mL). In particular, SEfr. 7 showed the most potent activity (95% inhibition at 30 μg/mL), which led us to investigate its active constituents preferentially. Fraction SEfr. 7 (15.4 g) was loaded onto a silica gel column (70 × 6 cm) and eluted with a *n*-hexane-EtOAc gradient (100:0, 95:5, 90:10, 85:15, 80:20, 70:30, 60:40, 40:60, 20:80, 0:100), and yielded another 13 subfractions (SEfr. 7-1 ~ 7-13). Silica gel column chromatography of SEfr. 7-4 and 7-5 that showed the highest activity (over than 90% inhibition at 15 μg/mL) led to the isolation of betulinic acid (**1**) [18] and betulinic acid methyl ester (**2**) [19] as active principles. Since betulinic acid (**1**) and its methyl ester **2** were detected in the fractions SEfr. 6, the activity of SEfr. 6 was regarded as the effect of compounds **1** and **2**. We tried to purify other active constituents from SEfr. 4 and 5. Column chromatography of SEfr. 4 (8.6 g) on a silica gel column (60 × 4.6 cm) using a gradient of *n*-hexane-EtOAc (100:0, 98:2, 96:4, 95:5, 92:8, 75:15, 70:30, 50:50, 30:70, 0:100) resulted in the purification of 1β-hydroxy arbusculin A (**8**, 28 mg) [20], dehydrocostuslactone (**4**, 1.6 g) [21], costunolide (**5**, 368 mg) [22], 11β,13-dihydrocostunolide (**6**, 49 mg) [23], and reynosin (**7**, 20 mg) [24]. SEfr. 5 (5 g) was also separated by MCI-gel CHP-20P column (60 × 5 cm) using a stepwise gradient of MeOH-H_2_O (10:90, 30:70, 50:50, 60:40, 70:30, 75:25, 90:10, 100:0), to give 12 fractions (SEfr. 5-1 ~ 5-12). Mokko lactone (**3**, 78 mg) [25] and α-costol (**10**, 87 mg) [26] were finally isolated from SEfr. 5-10 (1.0 g) through a silica gel column chromatography (50 × 4.3 cm) using *n*-hexane-CH_2_Cl_2_-EtOAc gradient system (20:0:0, 14:6:0, 14:6:0.5, 14:6:1, 14:6:2). Four other known compounds, santamarine (**9**) [24], β-sitosterol (**11**) [7], aplotaxene (**12**) [7], and α-amyrin stearate (**13**) [7] were also isolated during the purification process. The structures of compounds isolated (Figure 1) were determined by MS and NMR analysis, and confirmed by comparison of the spectroscopic data with those in literature [7].

### Assay method of PTP1B inhibitory activity

PTP1B (human, recombinant) was purchased from BIOMOL® International LP (Plymouth Meeting, PA). The enzyme activity was measured using *p*-nitrophenyl phosphate (*p*NPP) as a substrate [27]. To each of 96 wells in a microtiter plate (final volume: 100 μL) was added 2 mM *p*NPP and PTP1B (0.05-0.1 μg) in a buffer containing 50 mM citrate (pH 6.0), 0.1 M NaCl, 1 mM EDTA, and 1 mM dithiothreitol (DTT) with or without test compounds. Following incubation at 37°C for 30 min, the reaction was terminated with 10 M NaOH. The amount of produced *p*-nitrophenol was estimated by measuring the absorbance at 405 nm. The non-enzymatic hydrolysis of 2 mM *p*NPP was corrected by measuring the increase in absorbance at 405 nm obtained in the absence of PTP1B enzyme.
